# Parental Knowledge and Acceptance of Pediatric Lumbar Puncture in Northern Saudi Arabia: Implications for Clinical Practice and Education: A Cross-Sectional Study

**DOI:** 10.3390/pediatric17060129

**Published:** 2025-12-02

**Authors:** Dana Faez K. Alenezi, Rahaf Maqil T. Alanazi, Fai Fihat S. Almatrafi, Reema Mubarak O. Alanazi, Nouf Swilim K. Alenezy, Dalia Aqeel J. Alanazi, Shahad Wadi A. Alanazi, Rahaf Salman Z. Alanazi, Ayman Hamed Alenezi, Baraah Abu Alsel, Hanaa E. Bayomy, Safya E. Esmaeel, Manal S. Fawzy

**Affiliations:** 1 Faculty of Medicine, Northern Border University, Arar 91431, Saudi Arabia; dnoo1109@gmail.com (D.F.K.A.); rahafmagel112@gmail.com (R.M.T.A.); almatrafifai@gmail.com (F.F.S.A.); reema22r8@gmail.com (R.M.O.A.); nouf.swilim@gmail.com (N.S.K.A.); dalialsubaie1@gmail.com (D.A.J.A.); shooody441@gmail.com (S.W.A.A.); r0508073121@gmail.com (R.S.Z.A.); 2 Maternity and Children Hospital, Arar 73212, Saudi Arabia; ayhalenezi@moh.gov.sa; 3 Medical Sciences & Preparatory Year Department, North Private College of Nursing, Arar 73244, Saudi Arabia; baraahsel@nec.edu.sa; 4 Department of Family and Community Medicine, Faculty of Medicine, Northern Border University, Arar 43151, Saudi Arabia; hanaa.sayed@nbu.edu.sa; 5 Physiology Department, College of Medicine, Northern Border University, Arar 91431, Saudi Arabia; safya.ebraheem@nbu.edu.sa; 6 Center for Health Research, Northern Border University, Arar 73213, Saudi Arabia

**Keywords:** lumbar punctures, children, pediatric ages, awareness, Saudi Arabia

## Abstract

**Background/Objectives**: Lumbar puncture (LP) remains a vital pediatric procedure for diagnosing neurological and systemic conditions. Despite its clinical significance, parental hesitation to authorize pediatric LP often impedes early diagnosis and care. This study aims to evaluate parental knowledge and attitudes regarding pediatric LP in the Northern Border Region of Saudi Arabia, offering insights to inform targeted education strategies. **Methods**: A cross-sectional survey was conducted between February and August 2025 using a validated online questionnaire distributed via social media. The survey captured sociodemographic data and assessed awareness and attitudes toward pediatric LP. Univariate and multivariate logistic regression analyses examined factors associated with knowledge and consent. **Results**: Among 703 respondents, 60.6% were mothers and 95.6% were Saudi nationals. While 64.6% acknowledged the importance of aseptic technique, just 38.1% considered LP a safe practice. Knowledge levels were highest in parents aged 18–25 years (*p* < 0.001). Physician recommendation was the key factor in parental consent (87.0%), with 59.2% willing to approve the procedure following advice. Parents aged 26–35 years showed greater acceptance (OR = 1.54, 95% CI: 1.02–2.32, *p* = 0.04), whereas those older than 46 years were less receptive (OR = 0.51, 95% CI: 0.30–0.86, *p* = 0.01). **Conclusions**: Overall, parental knowledge regarding pediatric LP is limited. Targeted health education campaigns are needed to improve parental understanding of the procedure’s safety, importance, and benefits.

## 1. Introduction

Lumbar puncture (LP) has served as a cornerstone diagnostic and therapeutic procedure in medicine for over a century, with notable utility in the management of conditions of the central nervous system (CNS) [1]. By using a needle to access the subarachnoid space, clinicians collect cerebrospinal fluid (CSF) samples to facilitate the diagnosis of diseases such as subarachnoid hemorrhage, encephalitis, meningitis, and various neuroinflammatory or neoplastic disorders [2]. LP is routinely performed by specialists in pediatric emergency medicine, neurology, and pediatrics, especially when rapid CNS evaluation is required [3].

When conducted according to established protocols, LP is recognized for its favorable safety profile, with contraindications and major complications remaining relatively infrequent [4]. The most frequently encountered adverse events post-LP are headache, back pain, and infection. At the same time, rare but serious complications such as cerebral herniation, nerve injury, and paraplegia occur in select circumstances [5]. Recent studies indicate that proper procedural technique and patient selection can minimize these risks; some institutions have reported complication rates as low as 1% when protocol adherence is optimal [6,7].

Despite its proven clinical value and overall safety, parental acceptance of LP in pediatric populations is notably variable across regions and cultures [4,8,9,10,11,12,13,14]. In several Middle Eastern countries, including Saudi Arabia and the United Arab Emirates, refusal rates have been reported as high as 44%, with Kuwaiti parents refusing LP in up to 80% of cases [15,16,17]. These high-income countries face substantial barriers to acceptance, rooted in fear of complications, particularly paralysis, skepticism about the necessity of procedures, and a lack of trust in medical recommendations [18].

Recent cross-sectional studies in Saudi Arabia show that up to one-third of parents decline LP for their children, often citing anxiety regarding potential pain, paralysis, and misunderstanding of risks and benefits [14]. A survey among caregivers in the Western Region of Saudi Arabia highlighted that, while awareness of the purpose and aseptic technique of LP is generally good, significant gaps remain in knowledge regarding anesthesia, recovery time, and the true incidence of complications [19]. Educational attainment, clear physician communication, and trust in specialists are associated with higher parental acceptance [12].

Parental refusals can lead to delayed or missed diagnosis and suboptimal treatment of CNS diseases, with potentially serious consequences for affected children [4]. These consequences are especially pertinent in the Northern Border Region, where healthcare resources may be less centralized and access to specialized pediatric neurological services is limited compared to major urban centers in Saudi Arabia [20]. Parental hesitancy or refusal in this context may therefore have an even greater impact on diagnostic delays and clinical outcomes for children in the region. Understanding the specific knowledge gaps, sources of misconceptions, and decision-making processes among parents in this demographic is crucial for developing targeted educational initiatives and informed consent strategies that are regionally responsive. Prior research focusing on healthy individuals and family members of pediatric patients with febrile seizures repeatedly demonstrates that misconceptions regarding pain and catastrophic complications dominate the decision to refuse LP [19,21]. Standardizing the informed consent process and improving parental education are recommended strategies to address these hesitancies and improve uptake [12,22,23].

Given these region-specific challenges, the present study aims to comprehensively assess parents’ awareness and knowledge of pediatric LP in Saudi Arabia’s Northern Border Region, thereby providing context-specific baseline data to guide future educational interventions and healthcare policy development in pediatric neurological care.

## 2. Materials and Methods

### 2.1. Study Design and Setting

A descriptive cross-sectional study was conducted in the Northern Borders Region of Saudi Arabia from 12 February to 31 August 2025. This region comprises a diverse population with varying sociodemographic characteristics. The study targeted only Arabic-speaking parents of both genders aged 18 years and older who were residents of the Northern Borders Region at the time of data collection.

### 2.2. Inclusion Criteria

Eligible participants were parents (male or female) aged 18 years or older, residing in the Northern Borders Region, and able to read and understand Arabic (the official language of the participants). This ensured focus on the region-specific parental population and minimized external bias.

### 2.3. Sample Size Determination

Sample size was calculated using the Raosoft online sample size calculator (http://www.raosoft.com/samplesize.html) (accessed on 5 January 2025), taking into account the total population of the local region, a 95% confidence interval, a 5% margin of error, an expected response distribution of 50% to maximize requirements, and a default study power of at least 80%. An additional 5% was added to compensate for potential non-response, yielding a minimum sample size of 384. However, recruitment continued until 703 complete responses were obtained to enhance the statistical power for subgroup analyses, improve the precision of estimated proportions, reduce the margin of error, and increase the representativeness of diverse parental demographics within the region. This approach further strengthens the reliability and generalizability of our findings in this population.

### 2.4. Data Collection Tool and Questionnaire Development

Data were collected through a structured, self-administered electronic questionnaire (Supplementary File S1 Questionnaire-in-Arabic.pdf). All questionnaire items were set to mandatory in Google Forms, preventing the submission of incomplete surveys. The instrument was initially designed in Arabic to ensure linguistic appropriateness and cultural relevance. The questionnaire content was adapted from existing surveys, with guidance from relevant literature references [12,24]. The draft instrument underwent expert review by two board-certified pediatricians for content and face validity. Instrument reliability and clarity were further confirmed during a pilot with 20 parents, whose responses were excluded from the final analysis. The full questionnaire (Supplementary File S2 Questionnaire-in-English.pdf) comprised three sections:

Sociodemographic characteristics: age, gender, nationality, marital status, educational attainment, occupation, family income, and number of children.

Awareness and information sources: knowledge regarding pediatric LP, prior exposure to information, and trusted information channels. The level of knowledge about pediatric LP was assessed using nine questions. Participants responded to these questions as ‘yes’, ‘no’, or ‘I do not know’. Correct answers were coded as ‘1’ and wrong answers were coded as ‘0’. Responses were then summed to yield the knowledge score, ranging from 0 to 9. The knowledge score was then categorized as low (<33.33%, score < 4), moderate (33.33–66.66%; scores = 4–6), and high (>66.66%; scores > 6). The questionnaire and scoring system were previously validated in Saudi Arabia (Cronbach’s alpha = 0.718) [23].

Opinions and attitudes: willingness to approve or refuse LP for children, concerns regarding the procedure, and perceived risks and benefits.

### 2.5. Ethical Considerations

This study was conducted in accordance with the Declaration of Helsinki and its subsequent amendments. Ethical approval was obtained from the Bioethics Committee at Northern Border University, Saudi Arabia (HAP-09-A-043; Decision No. 18-25-H, dated 12 February 2025). All participants received detailed information regarding the study objectives, voluntary participation, and confidentiality protections. Informed electronic consent was obtained from each participant before the survey. Anonymity was strictly maintained, with no personally identifiable data collected or retained. Participants were free to withdraw at any point without penalty, and the study did not enroll any children.

### 2.6. Statistical Analysis

Data management and analysis were performed using STATA/SE version 11.2 (StataCorp, College Station, TX, USA) and Microsoft Excel. Descriptive statistics (means, standard deviations, frequencies, and percentages) were used to summarize participant characteristics and survey responses. Group comparisons for categorical variables were conducted using the Chi-squared (χ^2^) test or Fisher’s exact test, as appropriate. To assess relationships between parental sociodemographic factors, knowledge levels, and attitudes regarding pediatric LP, univariate and multivariable logistic regression analyses were performed. Odds ratios (OR) with 95% confidence intervals (CI) were calculated, and statistical significance was defined as a two-tailed *p*-value <0.05.

## 3. Results

A total of 703 parents were recruited, with mothers accounting for 60.60% (Table 1). The vast majority of parents were Saudi (95.59%), and the majority of parents were in the age groups 18–25 and 26–35 (32.57% and 32.43%, respectively). Those with more than 2 children accounted for 49.36%, with the highest proportions among parents being those who had graduated from university (82.50%) and were government employees (79.80%). Most parents reported medium income (60.46%), followed by high (26.17%), and low (13.37%).

**Figure 1:**
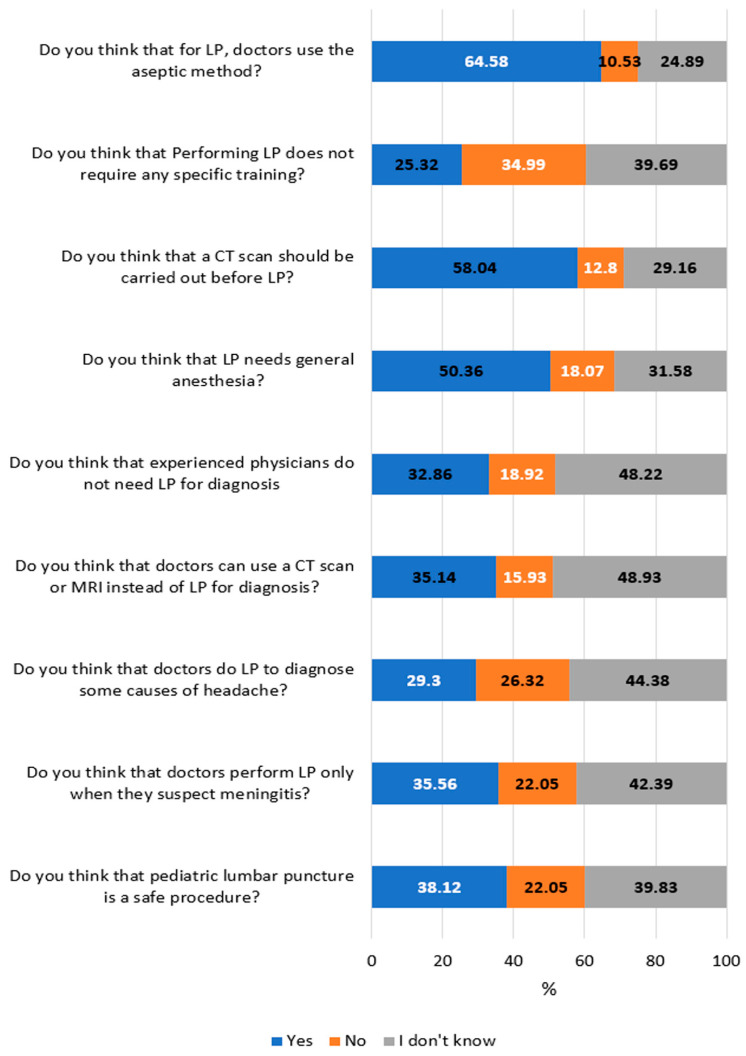
The frequency distribution of parents’ answers about their knowledge and awareness of pediatric lumbar punctures (n = 703). White percentages indicate correct answers.

Figure 1 illustrates parents’ responses to questions regarding their awareness and knowledge of pediatric LPs. Most parents know that doctors use an aseptic method for LP (64.58%), and only 38.12% thought that LP is a safe procedure. Regarding training to perform LP, 34.99% did not agree that LP does not require specific training. Two hundred and fifty parents reported that doctors perform LP only when they suspect meningitis, and two hundred and six parents thought that doctors perform LP to diagnose some types of headaches. Only 12.80% of respondents believed that a computed tomography (CT) scan should not be performed before LP. Additionally, 15.93% believed CT scans or MRIs could replace LP, 18.07% believed LP required general anesthesia, and 18.92% believed experienced physicians could diagnose without LP. The overall knowledge score ranged between 0 and 9, with low scores (the lower 33.33%, scores ≤ 3) accounting for 62.87%, moderate scores (33.33–66.66%, scores = 4–6) accounting for 34.57%, and the high scores (>66.66%, scores > 6) accounting for 2.56%.

**Figure 2:**
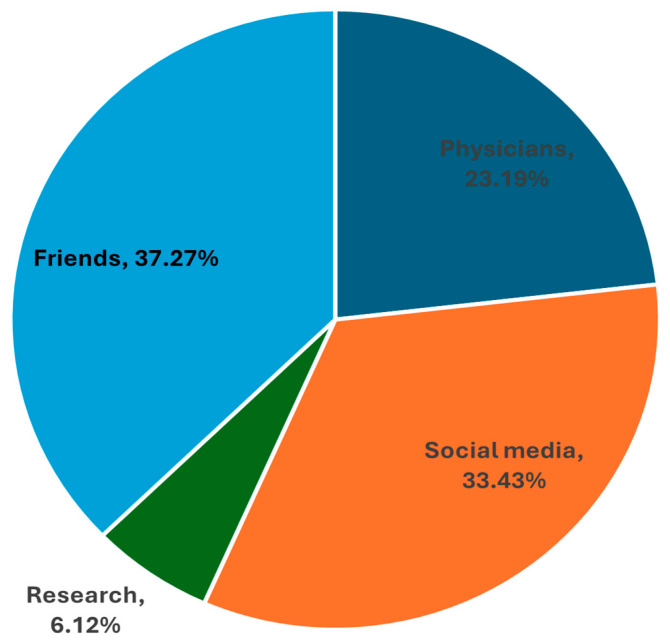
Reported sources of knowledge about pediatric lumbar puncture by the studied parents (n = 703).

Figure 2 shows that the most frequent source of information about pediatric LP was friends (37.3%), followed by social media (33.4%, physicians (23.2%), and research (6.1%).

Table 2 shows a significant association between parental age and knowledge about LP (χ^2^ = 18.00, *p* < 0.001). Parents aged 18–25 years were more likely to have moderate or high knowledge (OR = 1.98, 95% CI: 1.41–2.77), whereas those aged 36–46 years were less likely to have moderate or high knowledge (OR = 0.66, 95% CI: 0.45–0.98). Mothers were also less likely than fathers to have moderate or high knowledge (OR = 0.51, 95% CI: 0.37–0.71, *p* < 0.001). A significant association was also observed between LP knowledge and the number of children (χ^2^ = 71.39, *p* < 0.001). Parents with only one child were more likely to demonstrate moderate or high knowledge (OR = 3.93, 95% CI: 2.79–5.52), whereas those with more than two children were less likely (OR = 0.33, 95% CI: 0.24–0.46). Parental educational level was significantly associated with knowledge level (*p* = 0.04), with parents who completed secondary education more likely to have moderate or high knowledge (OR = 1.63, 95% CI: 1.02–2.58). Furthermore, household income was significantly associated with knowledge level. Parents with lower income were more likely to have moderate or high knowledge (OR = 2.24, 95% CI: 1.35–3.40), while those with higher income were less likely (OR = 0.58, 95% CI: 0.40–0.85).

The parents studied were asked whether they would consent to LP for their children (Table 3). More than half of the parents (n = 416, 59.17%) said they would consent to LP in their children following the physician’s advice (87.02%), for therapeutic (42.31%) and diagnostic reasons (33.89%). Meanwhile, 287 parents (40.83%) said they would refuse to consent to LP in their children because they believed the injection site was dangerous (82.23%), and they feared paralysis (80.49%) and death (78.05%).

A significant variation in parents’ attitudes towards pediatric LP was detected, based on parents’ age and household income (*p* < 0.001) (Table 4). Parents aged 26–35 years were more likely to consent to LP for their children (OR = 2.01, 95% CI: 1.42–2.86), whereas those aged 36–46 years (OR = 0.62, 95% CI: 0.43–0.90) and over 46 years (OR = 0.43, 95% CI: 0.26–0.71) were less likely to provide consent. Parents with medium household incomes were less likely to consent to LP (OR = 0.41, 95% CI: 0.29–0.58), while those with high incomes were more likely to do so (OR = 2.33, 95% CI: 1.59–3.45).

**Figure 3:**
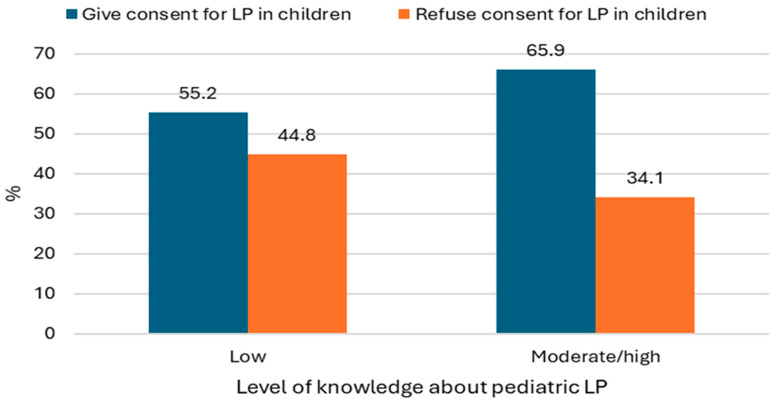
Relationship between parents’ awareness and knowledge about pediatric lumbar puncture and their opinions and attitudes towards accepting or refusing consent to lumbar puncture in children (n = 703; Chi-square test (χ^2^) = 7.77 and *p* = 0.005).

Parents who demonstrated moderate to high knowledge about pediatric LP were more likely to consent to LP in their children (*p* = 0.005) (Figure 3).

Table 5 presents the univariate and multivariate logistic regression analyses of parental knowledge and attitudes toward LP based on parental characteristics. In the multivariate model, parental age, type of parent, and household income remained significant predictors of LP knowledge. Moderate or high knowledge was less likely among parents aged 36–46 years (OR = 0.53, 95% CI: 0.34–0.83, *p* = 0.006) and those older than 46 years (OR = 0.47, 95% CI: 0.27–0.81, *p* = 0.006). Mothers were also less likely to have moderate or high knowledge compared with fathers (OR = 0.62, 95% CI: 0.44–0.87, *p* = 0.006). Additionally, parents with high household income were less likely to have moderate or high knowledge (OR = 0.48, 95% CI: 0.28–0.84, *p* = 0.01). For LP attitudes, parental age, household income, and LP knowledge remained significant predictors in the multivariate analysis. Parents aged 26–35 years were more likely to accept LP for their children (OR = 1.54, 95% CI: 1.02–2.32, *p* = 0.04), whereas those aged over 46 years were less likely to do so (OR = 0.51, 95% CI: 0.30–0.86, *p* = 0.01), compared with the 18–25-year age group. Parents with medium household income were less likely to accept LP (OR = 0.51, 95% CI: 0.31–0.84, *p* = 0.008) compared with those with low income. Additionally, parents with moderate or high knowledge about LP were more likely to accept the procedure for their children (OR = 1.60, 95% CI: 1.15–2.23, *p* = 0.006).

## 4. Discussion

Lumbar puncture remains an indispensable diagnostic and therapeutic procedure for several neurological disorders, facilitating the accurate identification and management of conditions such as meningitis and encephalitis [25]. Despite its clinical value and general safety when performed by trained individuals, parental hesitancy and refusal represent significant barriers in pediatric practice, as demonstrated both regionally and globally [4,11,22].

In this study, parental knowledge and acceptance of pediatric LP in the Northern Borders Region of Saudi Arabia were found to be low, consistent with results from other Saudi regions and international contexts [4,11,22]. Of the surveyed group, the majority were mothers (60.6%), a finding comparable to those in Western and Central Saudi Arabia [18,19]. Overall, 59.2% of parents indicated a willingness to consent to LP if recommended by a physician. In comparison, 40.8% would refuse, citing fears of complications, including paralysis and death, and viewing the procedure site as dangerous. These findings are consistent with previous studies, which also highlighted fear of paralysis (53.9%), procedural pain (68.2%), and concerns about insufficient information as key drivers of refusal [10,13].

Comparison with regional studies reveals substantial variability, likely due to differences in population exposure, educational attainment, and healthcare communication (Table 6). For instance, studies in Riyadh and Najran reported poor LP knowledge rates ranging from 41% to 56%, and a refusal rate of up to 44% [4,24]. However, it should be noted that the studies summarized in Table 6 varied significantly in methodology, including sampling approaches, target populations (e.g., parents vs. mothers only), questionnaire formats, and operational definitions for “low knowledge” and “refusal rate.” For example, some surveys relied on self-developed instruments, while others used previously validated tools; the thresholds for categorizing knowledge levels also differed. In addition, sample sizes and regional contexts varied. As such, direct quantitative comparisons between studies should be interpreted with caution, and this table primarily illustrates the variability in parental awareness and attitudes across settings rather than indicating precise inter-regional differences. Outside Saudi Arabia, Narchi et al. documented a 44.6% refusal rate in the UAE [16], with similar trends noted globally [11]. Sources of information for parents in this study skewed towards informal (friends), rather than professional (physicians), which may exacerbate misconceptions and anxiety regarding LP. Notably, physician recommendations were the strongest factor influencing acceptance, consistent with findings in other regions [12,18,19].

Demographically, lower LP knowledge was disproportionately observed among mothers, larger families, and households with higher incomes. This pattern requires further exploration, considering prior evidence linking education and trust in healthcare providers to improved knowledge and attitudes. Possible explanations for the potential association of higher household income with lower parental knowledge of pediatric LP, a finding that contrasts with most published literature where higher income or socioeconomic status generally predicts greater health knowledge and positive attitudes, may include differences in health information-seeking behaviors, trust in formal healthcare communications, or perhaps variations in exposure to pediatric neurological procedures within higher-income families in the present region. It is also possible that educational attainment, which is often more strongly associated with health knowledge, does not correlate directly with income in this population. Alternatively, our study’s reliance on convenience sampling and self-reported data may have introduced response biases affecting observed associations. This unexpected relationship invites further investigation through targeted qualitative research to clarify the underlying drivers of knowledge gaps in higher-income families [11,26].

Emerging patterns in our data suggest that parental trust in physician recommendations is a major determinant of LP acceptance. Possible reasons for variable trust may include physician nationality, age, years of experience, and perceived seniority, as highlighted in other regional studies [4,12]. In some cases, cultural concordance, communication style, and prior positive healthcare encounters have been shown to increase parental confidence and acceptance of procedures [27,28,29]. Conversely, lack of trust, potentially related to perceived junior status or insufficient experience, may contribute to heightened procedural anxiety and refusal [30]. These associations merit further exploration, as they may inform targeted recommendations on physician communication strategies and workforce training.

### 4.1. Clinical Implications and Future Directions

This study confirms persistent gaps in parental knowledge and prevalent negative perceptions of pediatric LP across Saudi regions, with direct consequences for the timely diagnosis and management of pediatric neurological diseases. Interventions such as educational outreach via social media, incorporation of tailored video tools, and training of pediatric staff in effective risk communication may substantially improve acceptance rates [30]. To facilitate implementation and guide stakeholders, Table 7 summarizes specific educational and outreach strategies, identifies the responsible actors, and highlights practical considerations and potential barriers. This structured overview is intended to support health authorities, clinicians, and policymakers in designing and prioritizing interventions tailored to the Saudi and Gulf regional context.

Future research should assess the longitudinal impacts of these interventions and expand to include diverse Saudi populations to enhance generalizability.

### 4.2. Limitations

This study has several important limitations. The geographic restriction to the Northern Borders Region may impact the applicability and generalizability of findings to other Saudi regions with distinct cultures and health beliefs. Additionally, only Arabic-speaking parents were eligible to participate, as the questionnaire was administered solely in Arabic; thus, non-Arabic-speaking parents were not represented, potentially further limiting generalizability.

A formal effect size calculation for subgroup analyses was not performed; although the sample is sufficiently powered for population estimation, future studies should consider method-specific power analyses. Also, reliance on online convenience sampling, with survey distribution via social media, introduces potential selection bias: parents with greater internet or health awareness may be overrepresented, thereby limiting representativeness. Because the survey was distributed via open social media channels, the total number of families approached and the response rate could not be determined. Non-responders may differ from responders in terms of internet access, health knowledge, or engagement, introducing additional selection bias and limiting generalizability.

Moreover, the cross-sectional, questionnaire-based design assesses parental knowledge and attitudes at a single point in time, precluding the assessment of temporal change or causal relationships. The scenarios presented in the survey were hypothetical; therefore, findings reflect parental intentions and self-reported attitudes, which may not necessarily predict actual consent behavior during real clinical encounters. While expert review and pilot testing were undertaken, some residual validation gaps may persist, and the questionnaire may not fully capture the multidimensional complexity of parental decision-making regarding LP.

Additionally, certain potential confounders, such as prior experience with LP (for their children or others), religious beliefs, trust in the healthcare system, or distance from healthcare facilities, were not assessed. These unmeasured variables may also influence parental knowledge and attitudes, and should be included in future studies for more comprehensive analysis.

These issues should be considered when interpreting our results, which are best viewed as associations rather than causally predictive findings. Future research using random sampling and direct observation of decision-making in clinical contexts is recommended to strengthen external validity and provide a more comprehensive understanding.

## 5. Conclusions

This study demonstrates that parental knowledge of pediatric LP in the Northern Borders Region of Saudi Arabia is generally low, with refusal driven primarily by fears of adverse outcomes such as paralysis and death. Physician recommendation remains pivotal in influencing parental consent. Efforts to improve public education and targeted communication are warranted to enhance understanding of LP’s clinical benefits and safety, advancing pediatric care and patient outcomes.

## Figures and Tables

**Table 1:** Sociodemographic characteristics of studied parents (n = 703).

Characteristic		No.	%
Age (year)	18–25	229	32.57
	26–35	228	32.43
	36–46	163	23.19
	>46	83	11.81
Nationality	Saudi	672	95.59
	Non-Saudi	31	4.41
Parents	Father	277	39.40
	Mother	426	60.60
Number of children	One child	254	36.13
	Two children	102	14.51
	>2 children	347	49.36
Parents’ educational status	University or higher	580	82.50
	Secondary school	93	13.23
	Preparatory school	12	1.71
	Primary school	11	1.56
	No educational certificate	7	1.00
Parents’ occupation	Governmental employee	561	79.80
	Private employee	78	11.10
	Unemployed	64	9.10
Household income	Low	94	13.37
	Medium	425	60.46
	High	184	26.17

Data are presented as numbers (No.) and percentages (%).

**Table 2:** Relationship between the level of awareness and knowledge about pediatric lumbar puncture and sociodemographic characteristics of studied parents (n = 703).

Characteristic		Low Knowledge (n = 442)		Moderate/High Knowledge (n = 261)		OR (95%CI)	χ^2^	*p*
		No.	%	No.	%			
Age (year)	18–25	119	26.92	110	42.15	1.98 (1.41–2.77) ***	18.00	<0.001
	26–35	151	34.16	77	29.50	0.81 (0.57–1.14)		
	36–46	114	25.79	49	18.77	0.66 (0.45–0.98) *		
	>46	58	13.12	25	9.58	0.70 (0.41–1.18)		
Nationality	Saudi	425	96.15	247	94.64	0.70 (0.32–1.57)	0.90	0.34
	Non-Saudi	17	3.85	14	5.36			
Parents	Father	148	33.48	129	49.43	0.51 (0.37–0.71) ***	17.46	<0.001
	Mother	294	66.52	132	50.57			
Number of children	One child	108	24.43	146	55.94	3.93 (2.79–5.52) ***	71.39	<0.001
	Two children	72	16.29	30	11.49	0.67 (0.41–1.07)		
	>2 children	262	59.28	85	32.57	0.33 (0.24–0.46) ***		
Parents’ educational status	University or higher	373	84.39	207	79.31	0.71 (0.47–1.07)	FET	0.04
	Secondary school	49	11.09	44	16.86	1.63 (1.02–2.58) *		
	Preparatory school	7	1.58	5	1.92	1.21 (0.30–4.49)		
	Primary school	6	1.36	5	1.92	1.42 (0.34–5.64)		
	No educational certificate	7	1.58	0	0.00	0 (0–0.92) *		
Parents’ occupation	Governmental employee	363	82.13	198	75.86	0.68 (0.46–1.01) *	4.01	0.13
	Private employee	43	9.73	35	13.41	1.44 (0.86–2.37)		
	Unemployed	36	8.14	28	10.73	1.35 (0.77–2.35)		
Household income	Low	44	9.95	50	19.16	2.14 (1.35–3.40) ***	16.60	<0.001
	Medium	266	60.18	159	60.92	1.03 (0.74–1.43)		
	High	132	29.86	52	19.92	0.58 (0.40–0.85) **		

χ^2^: Chi-square test; FET: Fisher’s exact test; OR (95% CI): Odds ratio (95% Confidence Interval); *p*: Probability, statistical significance was considered at *p* < 0.05. *: *p* < 0.05, **: *p*: < 0.01, ***: *p*: < 0.001.

**Table 3:** Reported reasons for consenting to or refusing LP in children by studied parents (n = 703).

Accept LP in Children (n = 416; 59.17%)			Refusing LP in Children (n = 287; 40.83%)		
Reasons *	No.	%	Reasons *	No.	%
Taking the advice of the doctor	362	87.02	Fear of paralysis	231	80.49
Possibly diagnostic	141	33.89	Injection site danger	236	82.23
Potentially therapeutic	176	42.31	Fear of death	224	78.05

Data are presented as numbers (No.) and percentages (%). * More than one answer was allowed.

**Table 4:** Relationship between parents’ opinions and attitudes towards pediatric lumbar puncture and their sociodemographic characteristics (n = 703).

Characteristics		Accept (n = 416; 59.17%)		Refusing (n = 287; 40.83%)		OR (95%CI)	χ^2^	*p*
		No.	%	No.	%			
Age (year)	18–25	140	33.65	89	31.01	1.13 (0.81–1.58)	28.5	<0.001
	26–35	160	38.46	68	23.69	2.01 (1.42–2.86) ***		
	36–46	82	19.71	81	28.22	0.62 (0.43–0.90) **		
	>46	34	8.17	49	17.07	0.43 (0.26–0.71) ***		
Nationality	Saudi	398	95.67	274	95.47	1.05 (0.46–2.30)	0.02	0.90
	Non-Saudi	18	4.33	13	4.53			
Parents	Father	159	38.22	118	41.11	0.89 (0.64–1.22)	0.59	0.44
	Mother	257	61.78	169	58.89			
Number of children	One child	157	37.74	97	33.80	1.19 (0.86–1.65)	2.37	0.30
	Two children	54	12.98	48	16.72	0.74 (0.48–1.16)		
	>2 children	205	49.28	142	49.48	0.99 (0.73–1.35)		
Parents’ educational status	University or higher	349	83.89	231	80.49	1.26 (0.83–1.90)	FET	0.75
	Secondary school	51	12.26	42	14.63	0.81 (0.51–1.30)		
	Preparatory school	7	1.68	5	1.74	0.96 (0.26–3.90)		
	Primary school	6	1.44	5	1.74	0.82 (0.21–3.45)		
	No educational certificate	3	0.72	4	1.39	0.51 (0.07–3.06)		
Parents’ occupation	Governmental employee	344	82.69	217	75.61	1.54 (1.04–2.27) *	5.99	0.05
	Private employee	42	10.10	36	12.54	0.78 (0.47–1.29)		
	Unemployed	30	7.21	34	11.85	0.58 (0.33–1.00) *		
Household income	Low	64	15.38	30	10.45	1.56 (0.96–2.56)	30.02	<0.001
	Medium	217	52.16	208	72.47	0.41 (0.29–0.58) ***		
	High	135	32.45	49	17.07	2.33 (1.59–3.45) ***		

χ^2^: Chi-square test; FET: Fisher’s exact test; OR (95% CI): Odds ratio (95% Confidence Interval); *p*: Probability, statistical significance was considered at *p* < 0.05. *: *p* < 0.05, **: *p* < 0.01, ***: *p* < 0.001.

**Table 5:** Logistic regression of parents’ knowledge and attitudes towards pediatric lumbar puncture (n = 703).

Characteristic	Level of Knowledge				Attitudes			
	Univariate Logistic Regression		Multiple Logistic Regression		Univariate Logistic Regression		Multiple Logistic Regression	
	OR (95%CI)	*p*	OR (95%CI)	*p*	OR (95%CI)	*p*	OR (95%CI)	*p*
Age (year)								
18–25	1.00		1.00		1.00		1.00	
26–35	0.55 (0.38–0.90)	0.002	0.69 (0.46–1.02)	0.06	1.49 (1.01–2.21)	0.04	1.54 (1.02–2.32)	0.04
36–46	0.46 (0.30–0.71)	<0.001	0.53 (0.34–0.83)	0.006	0.64 (0.43–0.96)	0.03	0.86 (0.56–1.33)	0.51
>46	0.47 (0.27–0.80)	0.005	0.47(0.27–0.81)	0.006	0.44 (0.26–0.73)	0.002	0.51 (0.30–0.86)	0.01
Nationality								
Saudi vs. non-Saudi	0.70 (0.34–1.46)	0.35			1.05 (0.50–2.18)	0.90		
Parents								
Mother vs. father	0.51 (0.38–0.70)	<0.001	0.62 (0.44–0.87)	0.006	1.13 (0.83–1.53)	0.44		
Number of children								
One child	1.00		1.00		1.00			
Two children	0.31 (0.19–0.50)	<0.001	0.31 (0.19–0.51)	<0.001	0.69 (0.44–1.10)	0.12		
>2 children	0.24 (0.17–0.34)	<0.001	0.26 (0.18–0.37)	<0.001	0.89 (0.64–1.24)	0.50		
Parents’ educational status								
University or higher	1.00				1.00			
Secondary school	1.62 (1.04–2.51)	0.03			0.80 (0.52–1.25)	0.33		
Preparatory school	1.29 (0.40–4.11)	0.67			0.93 (0.29–2.95)	0.90		
Primary school	1.50 (0.45–4.98)	0.51			0.79 (0.24–2.63)	0.71		
No educational certificate	Empty				0.50 (0.11–2.24)	0.36		
Parents’ occupation								
Governmental employee	1.00				1.00			
Private employee	0.70 (0.41–1.18)	0.18			1.80 (1.07–3.02)	0.03		
Unemployed	1.05 (0.54–2.04)	0.89			1.32 (0.68–2.56)	0.41		
Household income								
Low	1.00		1.00		1.00		1.00	
Medium	0.53 (0.33–0.82)	0.005	0.72 (0.45–1.17)	0.19	0.49 (0.30–0.78)	0.003	0.51 (0.31–0.84)	0.008
High	0.35 (0.21–0.58)	<0.001	0.48 (0.28–0.84)	0.01	1.29 (0.75–2.22)	0.36	1.23 (0.70–2.18)	0.47
Level of knowledge								
Moderate/high vs. low					1.57 (1.14–2.15)	0.005	1.60 (1.15–2.23)	0.006

OR (95% CI): Odds Ratio (95% Confidence Interval); *p*: Probability, statistical significance was considered at *p* < 0.05.

**Table 6:** Level of knowledge about pediatric lumbar puncture in different regions of Saudi Arabia.

Author/Year * [Reference]	Study Location	Sample Size	Reported Outcome(s)
Aldayel et al./2019 [18]	Riyadh	1223 questionnaires	Public ignorance about the LP technique foretells an unacceptably negative attitude toward the method.
Almatawah et al./2020 [17]	Al-Ahsa	466 participants	90% of participants had poor knowledge of LP, which was associated with a negative attitude towards LP.
Alshaibari, et al./2021 [4]	Najran	202 mothers	Of the respondents, four out of ten (40.6%) had never heard of LP. A significant minority of 89 mothers (44.0%) declined to give their kids LP.
Muammar NB et al./2022 [24]	Riyadh	1276 parents	56.1% had a bad perception, and 51.1% had poor knowledge of LP
Nemri et al./2025 [19]	Western Region of Saudi Arabia	993 participants	low knowledge in 75.5% of the participants.

* Author/year: first author surname/year of publication. All the studies included were cross-sectional surveys. Note: Studies included in this table differed in sampling methods, questionnaire content, definitions of knowledge/attitudes, and sample sizes; therefore, proportions may not be directly comparable.

**Table 7:** Proposed actionable strategies to improve parental acceptance of pediatric lumbar puncture.

Solution/Intervention	Key Actors	Implementation Approach	Practical Notes/Barriers
Physician-Led Parental Education	Physicians, Residents	Use clear pre-procedural counseling, standardized consent/education forms	Requires physician training in risk communication
Community Health Campaigns	Health authorities, NGOs	Social media, clinics, schools, culturally adapted leaflets, videos, Q&A events	Must address local language and misconceptions
Multidisciplinary Team Involvement	Nurses, Allied health staff	Nurses reinforce counseling, answer questions, and provide emotional support.	Coordination and engagement of non-physician staff are needed
Partnering with Local Community Leaders	Faith/community leaders	Include messages in religious gatherings, community events, and trusted influencers.	May facilitate trust and address cultural/religious gaps
Monitoring and Evaluation	Policymakers, Researchers	Assess impact, gather feedback, adapt approaches	Needs ongoing funding and stakeholder buy-in

## Data Availability

The original contributions presented in this study are included in the article/Supplementary Material. Further inquiries can be directed to the corresponding author.

## Supplementary Materials

The following supporting information can be downloaded at: https://www.mdpi.com/article/10.3390/pediatric17060129/s1, Supplementary File S1. Questionnaire-in-Arabic.pdf, Supplementary File S2. Questionnaire-in-English.pdf.

## Abbreviations

The following abbreviations are used in this manuscript:

CI	Confidence interval
CNS	Central nervous system
CSF	Cerebrospinal fluid
CT	Computed tomography
FET	Fisher’s exact test
LP	Lumbar puncture
No	Number
OR	Odds ratio
