# Supplementary material for: Parental Knowledge and Acceptance of Pediatric Lumbar Puncture in Northern Saudi Arabia: Implications for Clinical Practice and Education: A Cross-Sectional Study
Source: Pediatr Rep. 2025 Dec 2;17(6):129. doi: 10.3390/pediatric17060129 (PMC12735498; doi:10.3390/pediatric17060129)
Supplement: Supplementary file 1 [file pediatrrep-17-00129-s001.zip › Supplemnetary File S1_Questionnaire in Arabic.pdf]

الاستبيان بالعربي

العنوان : وعي الاباء تجاه سحب السائل الشوكي (الوخز اسفل الظهر) لدى الأطفال في منطقة الحدود الشمالية المملكة العربية السعودية

أ. المعلومات الشخصية

1	**العمر**<br><br>١٨ ـ ٢٥<br>٢٦ ـ ٣٥<br>٣٦ ـ ٤٥<br>أكثر من ٤٦
2	**الجنسية**<br><br>سعودي<br>غير سعودي
3	**الوالدان**<br><br>الأب<br>الأم
4	**عدد الأطفال**<br><br>طفل واحد<br>طفلان<br>أكثر من طفلين
5	**الحالة التعليمية للوالدين**<br><br>جامعي أو أعلى<br>مدرسة ثانوية<br>مدرسة إعدادية<br>مدرسة ابتدائية<br>لم يكمل تعليمه
6	**مهنة الوالدين**<br><br>موظف حكومي<br>موظف خاص<br>عاطل عن العمل
7	**دخل الأسرة**<br><br>منخفض<br>متوسط<br>مرتفع

ب. الوعي والمعرفة بسحب السائل الشوكي (الوخز أسفل الظهر) للمشاركين في الدراسة

١	هل تعرف عن سحب السائل الشوكي (الوخز أسفل الظهر) عند الأطفال؟	نعم<br>لا<br>لا أعرف
٢	هل تعتقد أن سحب السائل الشوكي (الوخز أسفل الظهر) عند الأطفال إجراء آمن؟	نعم<br>لا<br>أعرف لا
٣	هل تعتقد أن الأطباء يقومون بسحب السائل الشوكي (الوخز أسفل الظهر) فقط عندما يشتبهون في التهاب السحايا؟	نعم<br>لا<br>أعرف لا
٤	هل تعتقد أن الأطباء يقومون بسحب السائل الشوكي (الوخز أسفل الظهر) لتشخيص بعض أسباب الصداع؟	نعم<br>لا<br>أعرف لا
٥	هل تعتقد أن الأطباء يمكنهم استخدام التصوير المقطعي المحوسب أو التصوير بالرنين المغناطيسي بدلاً من سحب السائل الشوكي (الوخز أسفل الظهر) للتشخيص؟	نعم<br>لا<br>أعرف لا
٦	هل تعتقد أن الأطباء ذوي الخبرة لا يحتاجون إلى سحب السائل الشوكي (الوخز أسفل الظهر) للتشخيص ؟	نعم<br>لا<br>أعرف لا
٧	هل تعتقد أن سحب السائل الشوكي (الوخز أسفل الظهر) يحتاج إلى تخدير عام؟	نعم<br>لا<br>أعرف لا
٨	هل تعتقد أنه يجب إجراء التصوير المقطعي المحوسب قبل سحب السائل الشوكي (الوخز أسفل الظهر)؟	نعم<br>لا<br>أعرف لا
٩	هل تعتقد أن إجراء سحب السائل الشوكي (الوخز أسفل الظهر) لا يتطلب أي تدريب محدد	نعم<br>لا<br>أعرف لا
١٠	هل تعتقد أن الأطباء يستخدمون الطريقة المعقمة لسحب السائل الشوكي (الوخز أسفل الظهر) ؟	نعم<br>لا<br>أعرف لا

١١	ماهي مصدر معلوماتك	الأطباء<br>وسائل التواصل الاجتماعي<br>الأبحاث<br>الأصدقاء

د. آراء ومواقف المشاركين في الدراسة تجاه سحب السائل الشوكي(الوخز أسفل الظهر) للأطفال

1	مامدى استجابتك لإجراء سحب السائل الشوكي (الوخز أسفل الظهر) عند الأطفال ؟	قبول<br>رفض
2	إذا قبلت بسحب السائل الشوكي (الوخز أسفل الظهر) لطفلك ما هي الأسباب؟	اتباع نصيحة الأطباء<br>لتشخيص محتمل<br>لعلاج محتمل
3	إذا رفضت سحب السائل الشوكي (الوخز أسفل الظهر) لطفلك ما هي الأسباب؟	موقع الحقنة خطير<br>الخوف من الموت<br>الخوف من الشلل

Questionnaire references

1. Muammar NB, Rohaimi NA, Aleid B, Harbi AA, Yousif A. Level of awareness of parents toward pediatric lumbar punctures in Riyadh, Saudi Arabia. SJEMed. 2020; 1(2): 96-102. doi: 10.24911/SJEMed/72-1586249695.
2. Sahin A, Kara-Aksay A, Demir G, Ekemen-Keles Y, Ustundag G, Berksoy E, Karadag-Oncel E, Yilmaz D. Parental Attitudes About Lumbar Puncture in Children With Suspected Central Nervous System Infection. Pediatr Emerg Care. 2023 Sep 1; 39(9):661-665. doi: 10.1097/PEC.0000000000003015. Epub 2023 Jul 19. PMID: 37463198.
