# Supplementary material for: Parental Knowledge and Acceptance of Pediatric Lumbar Puncture in Northern Saudi Arabia: Implications for Clinical Practice and Education: A Cross-Sectional Study
Source: Pediatr Rep. 2025 Dec 2;17(6):129. doi: 10.3390/pediatric17060129 (PMC12735498; doi:10.3390/pediatric17060129)
Supplement: Supplementary file 1 [file pediatrrep-17-00129-s001.zip › Supplemnetary File S2_Questionnaire in English.pdf]

## Questionnaire in English

**Title: Parental Knowledge and Acceptance of Pediatric Lumbar Puncture in Northern Saudi Arabia: Implications for Clinical Practice and Education: A Cross-Sectional Study**

### A-Personal information

| | |
|---|---|
| 1. | Age<br><br>18-25<br>26-35<br>36-46<br>More than 46 |
| 2. | Nationality<br><br>Saudi<br>Non-Saudi |
| 3. | Parents<br>Father<br>Mother |
| 4. | Number of Children<br>One child<br>Two children<br>More than two children |
| 5. | Parents' Educational status<br><br>University or higher<br>Secondary school<br>Preparatory school<br>Primary school<br>Illiterate |
| 6. | Parents' Occupation<br><br>Government employee<br>Private employee<br>Unemployed |
| 7. | Household income<br>Low<br>Medium<br>High |

### B. Awareness and knowledge about LP among the study participants

| | | |
|---|---|---|
| 1 | Do you know about pediatric lumbar puncture? | Yes<br><br>No<br><br>I don't know |

| | | |
|---|---|---|
| 2. | Do you think that pediatric lumbar puncture is a safe procedure? | Yes<br>No<br>I don't know |
| 2 | Do you think that doctors perform LP only when they suspect meningitis? | Yes<br>No<br>I don't know |
| 3 | Do you think that doctors perform LPs to diagnose some causes of headaches? | Yes<br>No<br>I don't know |
| 4 | Do you think that doctors can use a CT scan or MRI instead of LP for diagnosis? | Yes<br>No<br>I don't know |
| 5 | Do you think that experienced physicians do not need LP for diagnosis | Yes<br>No<br>I don't know |
| 6 | Do you think that LP needs general anesthesia? | Yes<br>No<br>I don't know |
| 7 | Do you think that a CT scan should be carried out before LP? | Yes<br>No<br>I don't know |
| 8 | Do you think that Performing LP does not require any specific training? | Yes<br>No<br>I don't know |
| 9 | Do you think that for LP, doctors use the aseptic method? | Yes<br>No<br>I don't know |
| 10 | Source of your information | Physicians<br>Social media<br>Researches<br>Friends |

**D. Opinions and attitudes of participants toward Pediatric lumbar puncture**

| | | |
|---|---|---|
| 1. | Your response to the lumbar Puncture procedure in children | Accept<br>Refuse |
| 2. | If you accept, What are the reasons? | Following the doctors' advice<br>Potentially diagnostic<br>Potentially therapeutically |
| 3. | If you refuse, what are the reasons? | Injection site danger<br>Fear of death<br>Fear of paralysis |

**Questionnaire reference:**


1. Muammar NB, Rohaimi NA, Aleid B, Harbi AA, Yousif A. Level of awareness of parents toward pediatric lumbar punctures in Riyadh, Saudi Arabia. SJEMed. 2020; 1(2): 96-102. doi: 10.24911/SJEMed/72-1586249695.
2. Sahin A, Kara-Aksay A, Demir G, Ekemen-Keles Y, Ustundag G, Berksoy E, Karadag-Oncel E, Yilmaz D. Parental Attitudes About Lumbar Puncture in Children With Suspected Central Nervous System Infection. Pediatr Emerg Care. 2023 Sep 1;39(9):661-665. doi: 10.1097/PEC.0000000000003015. Epub 2023 Jul 19. PMID: 37463198.
